# Scaling up Quality Improvement for Surgical Teams (QIST) – avoiding surgical site infection and anaemia at the time of surgery: protocol for a cluster randomised controlled trial

**DOI:** 10.1186/s13063-020-4152-3

**Published:** 2020-02-28

**Authors:** Ashley B Scrimshire, Alison Booth, Caroline Fairhurst, Mike Reed, Win Tadd, Annie Laverty, Belen Corbacho, David Torgerson, Catriona McDaid

**Affiliations:** 1 Department of Health Sciences, University of York, Heslington, York, YO10 5DD UK; 2 Northumbria Healthcare NHS Foundation Trust, Woodhorn Lane, Ashington, NE63 9JJ UK; 3 Cardiff, UK

**Keywords:** Implementation at scale, Anaemia, Surgical site infection, Surgery, Breakthrough series collaborative

## Abstract

**Background:**

Measures shown to improve outcomes for patients often fail to be adopted into routine practice in the NHS. The Institute for Health Improvement Breakthrough Series Collaborative (BSC) model is designed to support implementation at scale. This trial aims to assess the effectiveness and cost-effectiveness of quality improvement collaboratives (QICs) based on the BSC method for introducing service improvements at scale in the NHS.

**Methods:**

Forty Trusts will be randomised (1:1) to introduce one of two protocols already shown to improve outcomes in patients undergoing elective total hip and knee replacement surgery.

The intervention is improvement collaboratives based on the BSC model, a learning system that brings together a large number of teams to seek improvement focussed on a proven intervention. Collaboratives aim to deliver at scale, maximise local engagement and leadership and are designed to build capacity, enable learning and prepare for sustainability. Collaboratives involve Learning Sessions, Action Periods, and a summative congress.

Trusts will be supported to introduce either: decolonisation for Methicillin Sensitive *Staphylococcus aureus* (MSSA) to reduce post-operative infection (QIST: Infection), or an anaemia optimisation programme to reduce peri-operative blood transfusions (QIST: Anaemia). Trusts will continue with their usual practice for whichever protocol they are not introducing. Anonymised data related to both infection and anaemia outcomes for patients undergoing hip or knee arthroplasty at all sites will mean that the two groups act as controls for each other.

The primary outcome for the QIST: Infection collaborative is deep MSSA surgical site infection within 90 days of surgery, and for the QIST: Anaemia collaborative is blood transfusion within 7 days of surgery. Patient-level secondary outcomes include length of hospital stay and readmission, which will also inform the economic costings. Qualitative interviews will evaluate the support provided to teams.

**Discussion:**

The scale of this trial brings considerable challenges and potential barriers to delivery. Anticipated challenges relate to recruiting and sustaining up to 40 organisations, each with its own culture and context. This complex project with multiple stakeholders across a large geographical area will be managed by experienced senior-level project leaders with a proven track record in advanced project management. The team should ensure effective project governance and communications.

**Trial registration:**

ISRCTN, ISRCTN11085475. Prospectively registered on 15 February 2018.

## Background

Across all of healthcare there are known gaps between what the evidence shows to be best practice and the care that patients receive. The reasons for this are often complex and multifactorial. Efforts to improve quality show mostly inconsistent and patchy results [1–3].

Quality improvement programmes can provide a framework to help bridge the evidence-to-practice gap. One technique is a quality improvement collaborative (QIC). The general aim of any QIC is to introduce change at scale and at pace by encouraging collaboration between teams from different healthcare systems. The specific clinical process, pathway or outcome being targeted can vary widely as can the healthcare setting in which these are being implemented. The Institute for Healthcare Improvement (IHI) developed the Breakthrough Series Collaborative (BSC) methodology as one way to design and deliver an improvement collaborative.

A collaborative is a short-term (6- to 15-month) learning system that brings together a large number of teams from hospitals or clinics to seek improvement in a focussed topic area. Collaboratives range in size from 12 to 160 organisational teams [4]. Each team typically sends three of its members to attend Learning Sessions (face-to-face meetings) over the course of the collaborative, with additional members working on improvements in the local organisation [4]. Teams in such collaboratives have achieved dramatic results, including reducing waiting times by 50%, reducing worker absenteeism by 25%, reducing intensive care unit costs by 25%, and reducing hospitalisations for patients with congestive heart failure by 50% [4]. However, this model is yet to be tested at scale in the English National Health Service (NHS).

A systematic review of the existing evidence on QICs highlighted the need for randomised controlled trials (RCTs) to assess their effects on the process of care, and provider- and patient-level outcomes [5]. In particular the review identified a lack of evidence on whether the procedural improvements associated with collaboratives translate into patient-level outcomes. In a more recent review of the effectiveness of QICs, Wells et al. (2017) found some encouraging results [6]. However, the authors also highlight the need to address significant, persistent gaps in QIC design, quality of reporting, sustainability and cost-effectiveness. A recent report from New Zealand implies that BSCs can work within elective joint replacement centres, although the effect could not be separated from a natural improvement in healthcare standards, as was happening prior to the intervention, in part due to the before-and-after study design [7]. These studies illustrate the complex nature of introducing any change in practice and reinforce the need for further high-quality evidence. The existing evidence demonstrates the feasibility and potential effectiveness of QICs and provides valuable insights for study design and outcome measures.

## Aims and objectives

The aim of the trial is to assess the clinical- and cost-effectiveness of QICs, based on IHI BSC methodology, to implement large-scale change in the NHS, specifically for improving outcomes in patients undergoing elective total hip and knee joint replacement. To achieve this, we will compare the roll-out of two different improvement initiatives, each initiative focussing on a different pre-operative measure for improving post-operative outcomes. These are, pre-operative anaemia management (QIST: Anaemia) and pre-operative Methicillin Sensitive *Staphylococcus aureus* (MSSA) decolonisation (QIST: Infection).

The trial objectives are to measure patient-level outcomes such as transfusion and infection rates, length of stay in hospital, readmission rates and critical care admissions; and process-of-care measures related to compliance with elements of the quality improvement protocols.

### QIST: Anaemia, implementing pre-operative anaemia management

Pre-operative anaemia in patients undergoing elective hip and knee replacement is associated with increased post-operative morbidity and mortality, as well as increased red blood cell transfusion rates, hospital readmissions and a longer length of stay [8]. Patient Blood Management (PBM) is a multidisciplinary approach which aims to optimise the care of patients who may require a blood transfusion. A key element of this involves screening for, and correcting, anaemia and/or iron deficiency pre-operatively. UK National and International Guidance recommends the optimisation of anaemia prior to surgery [9–11]. However, it is recognised that implementation of pre-operative anaemia pathways is challenging, leading to varying rates of their implementation [12, 13].

In the UK the 2015 National Comparative Audit of Blood Transfusion was performed in 190 hospitals and stated that hospitals should have a pre-operative management protocol which allows for timely identification and treatment of anaemia before elective surgery [14]. They concluded that there is a need to increase the investigation and management of pre-operative anaemia in the UK. They stated that improvement in practice to help to ensure appropriate use of transfusion and alternatives would benefit patients and reduce healthcare costs. There are examples of similar initiatives in Europe, the United States and Australia [11, 15, 16].

The QIST: Anaemia arm of the trial aims to support teams in developing and implementing pre-operative anaemia-screening and -management pathways in their local Trust through the use of a QIC. It is expected that this will lead to a reduction in the number of patients requiring peri-operative blood transfusion, the number of units transfused and a reduced length of inpatient stay. There is mixed evidence on the impact of anaemia management on critical care admission and emergency hospital readmission rates, and these will also be assessed in this trial.

### QIST: Infection, implementing pre-operative Methicillin Sensitive *Staphylococcus Aureus* (MSSA) decolonisation

Total joint replacement (TJR) is increasing year on year with an ageing population, with 252,251 cases being recorded in the UK National Joint Registry for the year 2017/2018 [17]. Surgical site infection (SSI) is a serious and life-threatening complication of a TJR. Estimates of SSI rates vary between 1 and 5% with the true rate likely to be around 3% and 3.3% for total hip and knee replacements, respectively [18]. Infection in a TJR can result in prolonged antibiotic use, repeat operations and revision surgery as well as fusion of the joint and amputation in rare cases [19]. Patients who develop infection often have a poor outcome, even when the infection has been cleared. There is a heavy long-term burden on the patient and deep infections have a higher mortality rate than prostate, breast and colorectal cancer at 5 years [20]. Each deep infection costs up to £75,000 to treat and, scaled up, the NHS cost of TJR SSI is approximately £45 million per annum, based upon an average cost of £10,000 per infection [21, 22].

Multiple systematic reviews and meta-analyses of *Staphylococcus aureus* screening and decolonisation in orthopaedic surgery have found this to be a cost-effective method to reduce SSIs [23, 24]. In December 2016 the World Health Organisation recommended decolonisation of patients with nasal carriage of *S. aureus* undergoing orthopaedic surgery, although this still rarely occurs within the NHS [25].

The QIST: Infection arm of the trial aims to support teams in developing and implementing pre-operative MSSA screening and/or decolonisation pathways in their local Trust through the use of a QIC. It is expected that this will lead to a reduction in the number of patients suffering a post-operative SSI (deep and superficial).

### Anaemia and infection relationship

The trial assumes that the two primary outcomes, blood transfusion and SSI, are independent and that the intervention implemented in one trial arm does not affect the outcome of the other, i.e. MSSA decolonisation does not affect transfusion rates, and anaemia screening does not affect SSI rates.

It is safe to assume that reducing SSIs would not affect the rate of blood transfusion within 7 days of primary surgery. SSIs typically take longer than this to develop and, as such, any further surgery, i.e. revision arthroplasty, which may increase the risk of transfusion, would be after this time. In one study the minimum time from primary surgery to diagnosis of SSI was 11 days [26].

Conversely, there is evidence that being anaemic increases the risk of a range of post-operative complications, including infection, and that improving anaemia before surgery can reduce the risk of some of these complications [8, 11]. However, what has not been established is whether optimising anaemia pre-operatively reduces post-operative SSI risk. It is theoretically possible that by improving anaemia in the QIST: Anaemia arm of the trial we also reduce infection rates; however, there is no evidence base for this from existing clinical trials. In addition, it is expected that the effect size of MSSA decolonisation in reducing SSI will be large enough to still demonstrate a difference.

Iron treatment is often indicated to correct pre-operative anaemia, this can be given orally or intravenously [11]. There has been some concern that the use of intravenously administered (IV) iron may potentially increase infection rates as iron is a good medium for bacterial growth. However, a systematic review of clinical trials has deemed there to be no evidence supporting this theory [27]. In addition, it is anticipated that most patients requiring iron treatment will take orally administered iron, with only a minority requiring IV iron [28]. Therefore, this is assumed to not be relevant to the QIST trial.

### Rationale

Both pre-operative protocols are feasible and the purpose of this cluster RCT is to establish whether they can be introduced at pace and at scale in the English NHS using a QIC approach. Trusts will be randomised to implement one of the two initiatives for the duration of the trial. Between Learning Sessions, teams will test and implement changes in their local settings and collect data to measure the impact change, supported by learning and quality accounts. The Model for Improvement will be applied as a way of testing small-scale improvement cycles [4]. Following completion of the study period, all Trusts will be given the opportunity to be trained in, and implement, the alternate quality improvement initiative.

The projected saving across 30,000 joint replacements at 40 Trusts from the anaemia-screening initiative is £4.8 million. From training 40 Trusts in MSSA screening and decolonisation it is anticipated that infection will be avoided in 0.5% of 30,000 joints, each costing an average of £10,000, making a projected saving of £1.5 million. The cost savings are in addition to the reduction in pain, distress, mobility and morbidity issues for patients who would otherwise have suffered these complications.

The choice of primary total hip and knee replacement surgery for the trial is supported by the fact that these are standard operations routinely undertaken in sizable numbers in most NHS Trusts in England. There is existing supporting data for the planned protocols relevant to total hip and knee replacement surgery, demonstrating the potential for scalable effects across the NHS. Unicompartmental knee replacements and hip resurfacing have been excluded as they are associated with lower transfusion and infection rates compared to total hip and knee replacements [29–32].

## Methods

### Study design

We will use a cluster randomised design which is the most robust method to establish whether outcomes are attributable to the quality improvement initiative, rather than a secular trend. A RCT allows the control of known and unknown variables in order that a causal relationship can be established between an intervention and outcome [33]. Because the quality improvement initiatives that we are evaluating are targeted at healthcare teams, it will be necessary to use a cluster RCT whereby Trusts will be randomly allocated to one of the two groups. The two study objectives related to anaemia and MSSA will be addressed within a single RCT. The fact that all hospitals will receive a quality improvement initiative will enable us to address the ‘Hawthorne effect’ of taking part in an implementation/research project [34]. So, any difference in outcomes that we observe can be attributed to the intervention rather than an ‘observation’ effect.

### Study setting, population and recruitment

Working with NHS Improvement and the British Orthopaedic Association (BOA) we aim to recruit and randomise 40 of the 139 acute NHS Trusts in England performing elective total hip or knee replacement surgery. Recruitment of sites will be through direct correspondence with acute Trust chief executive officers and clinical teams.

### Eligibility and exclusion criteria

All English NHS acute Trusts performing elective primary total hip and/or total knee arthroplasty are eligible. Trust executives must commit to providing consent to participate if a Trust is recruited, as executive support for the project form an early stage will be essential.

Trusts will be excluded if either it is already routine practice for orthopaedic surgical patients to be screened and/or managed for pre-operative anaemia or to be screened and/or decolonised for MSSA.

### Screening and pre-randomisation procedures

Trusts expressing an interest in participating will be assessed against the inclusion and exclusion criteria for eligibility.

If more than 40 NHS Trusts express an interest in taking part in the study and are identified as suitable for inclusion, selection will preference those performing a greater annual number of hip and knee replacement procedures, as reported by the National Joint Registry (NJR) [35].

Trusts identified as suitable for inclusion will be informed and Trust-level consent to participate obtained.

### Enrolment procedure

A contract between the sponsor and each participating site, setting out the responsibilities of the sponsor, chief investigator (CI) and site, including site principal investigator (PI), will be in place. The contract will include research permissions, clear governance and measurement and communications protocols which will also help build engagement and enthusiasm. An academic lead and project manager will be identified for each site.

### Sample size

There are limited published data on the specific rates of post-operative MSSA SSI in England following a total hip or knee replacement. In a large, randomised multicentre trial, the risk of developing hospital-associated *Staphylococcus aureus* infection in MSSA-carrier patients who were decolonised on admission to hospital (using mupirocin nasal ointment and chlorhexidine soap) fell from 7.7 to 3.4% [36]. In a retrospective cohort study, performed by Northumbria Healthcare NHS Foundation Trust, a decrease in MSSA infection rate from 0.75% (28/3593) to 0.25% (23/9318) was found following the adoption of an MSSA decolonisation programme for carriers of MSSA in elective joint replacement [37]. To detect a difference from 0.75 to 0.25% would require 6246 patients in an individually randomised trial with 80% power. Between October 2015 and September 2016, 123,861 hip and knee operations were undertaken in 139 hospital Trusts (average 891) [38]. Assuming an intraclass correlation coefficient (ICC) of 0.005 [39], and a more conservative average of 750 patients per site, we require 40 sites to be recruited and randomised in this cluster randomised trial.

Blood transfusion rates vary widely between hospitals. Estimates suggest that hospitals’ transfusion rates in the period from 28 days before surgery until 14 days post-operatively vary from < 10 to > 90% for total hip replacement, with an overall rate of 25% [14]. Eighteen percent were transfused in the post-operative period from 24 h to 14 days post-operatively [14]. For total knee replacement, hospitals’ transfusion rates vary from 0 to 39% (overall 19%) [40]. In routine total hip arthroplasty and total knee arthroplasty, the prevalence of allogeneic red blood cell transfusions has been reported to be between 21 and 70%, with the majority of authors reporting figures in the middle of the range [41].

In a prospective comparative cohort study of patients from a single site who underwent elective hip and knee arthroplasty before (control) and after (intervention) the launch of the anaemia optimisation programme, it was found that 3.9% (63/1622) of patients required an allogeneic red blood cell transfusion within 30 days following surgery in the intervention group and 6.0% (108/1814) in the control group [28]. With 40 sites, assuming an ICC of 0.005, and an average of 750 patients per site, we would have over 95% power to detect this difference in transfusion rates.

Therefore, our sample size is for 40 Trusts, each undertaking an average of 750 procedures per annum and will be randomised on a 1:1 ratio. This sample is powered to detect the smaller of the expected differences, a change in SSI rates.

### Randomisation

Each participating NHS Trust will be treated as a cluster and will be randomised 1:1 using minimisation by number of hip and knee replacement procedures performed in a 12-month period, as reported by the NJR, (cut at the median) and the traffic-light indicators in the Learning From Mistakes league table (outstanding/good/significant concerns/poor) [42]. Minimisation will be via the dedicated desktop application programme, MinimPy [43]. Trusts will be randomised to receive either training on MSSA decolonisation to control post-operative infection or training on the anaemia optimisation programme. The control group for the anaemia optimisation quality improvement initiative will be the other hospitals which will continue with their usual practice for anaemia and vice versa, the control group for MSSA will be the other hospitals who will continue with their usual practice for MSSA. Each Trust will be issued with a unique trial site identification number at randomisation.

To minimise contamination or resentful demoralisation between the different quality improvement initiative groups, which would reduce the possibility of detecting important change (for example, an anaemia initiative hospital trying to improve MSSA screening at the same time), hospitals will be given the opportunity to be trained in the quality improvement initiative that they have not received after the evaluation period is over.

### Blinding

Trusts and their nominated clinicians will be informed of the quality improvement initiative to which they have been randomised. It will not be possible to blind Trusts or treating clinicians to the collaborative intervention or their allocated quality improvement initiative. However, the clinical team will take no part in the quantitative assessment process. The functional outcome data will be collected by Trust information teams and passed directly to an independent company (e-Dendrite) for merging and anonymisation.

Aggregated data will be fed back to Trusts as part of the intervention process. A procedure for breaking codes or un-blinding is, therefore, not necessary.

### Intervention

The study intervention is a QIC based on the IHI BSC model [4]. A collaborative is a short-term learning system that brings together a large number of teams to seek improvement in a focussed topic area and proven intervention. Collaboratives provide a suitable vehicle for delivery of a project at this scale, as they not only aim to maximise local engagement and leadership but are designed to build capacity, enable learning and prepare for sustainability. The following intervention description is in line with the TIDieR guidelines [44]. It is possible that what is ultimately delivered may differ as the collaborative programme evolves based on the needs and feedback from participating sites. TIDieR and SQUIRE guidelines will be used to assist with detailed reporting of the intervention delivered in the final report [44, 45].

Key elements of the collaborative are detailed below and summarised in Table 1.

**Table 1:** Summary of how elements of Institute for Health Improvement Breakthrough Series Collaborative (IHI BSC) will be applied to the Quality Improvement for Surgical Teams (QIST) trial

Elements of collaborative	Planned approach/rationale for this study
Topic selection	Real-world improvements seen with these MSSA and anaemia-screening protocols
	They are not yet widely used across the NHS
Faculty recruitment	CI and clinical lead trained in BSC methodology
	Team of experts recruited to help guide project and advise teams at learning events
Enrolment of teams	Calls for interested centres through BOA and NHS Improvement to all NHS Trusts in England to senior leaders, management and clinicians to increase support and engagement
	Selection procedure if more than 40 interested Trusts identified
	All team members to be healthcare professionals and ideally GCP trained but this is not essential
Learning Sessions	Separate dates for anaemia and MSSA learning sessions to avoid contamination
	3 x 1-day, face-to-face group Learning Sessions per group
	Attendees: experts, programme leads, improvement fellows, patient leaders and four study team members from each Trust
	Content: teach Trusts the relevant protocol, review evidence, governance arrangements, business cases, communications, pathways, data collection and reporting arrangements
	Further series of 3 Learning Sessions at the end of the study period to teach all Trusts both interventions
Action Periods	Local teams implement change and collect data to measure the impact of change
	Bespoke electronic data collection system will be developed and maintained throughout the study
	Monthly progress reports will be sent to Trusts including number or operations performed compared to expected activity
	Learning Sessions will act as networking events for collaborative working and problem-solving
	Study team members will be contactable for further advice as required
Summative congress and publications	Initial summative session at the end of the 1st round of implementation for each separate trial arm
	Final summative session to be held with all teams and collaborators invited
	Results to be presented at BOA Congress 2020
	Publication in high-impact journal will be sought
	Potential influence on national guidelines
Measurement and evaluation	Bespoke IT system will automatically generate near-to-live run charts and improvement metrics for individual teams and the collaborative as a whole so that teams and faculty can track progress over time

*BOA* British Orthopaedic Association, *BSC* Breakthrough Series Collaborative, *IT* information technology, *NHS* National health Service, *MSSA* Methicillin Sensitive *Staphylococcus aureus*

### Topic selection

*‘Leaders identify a particular area or issue in healthcare that is ripe for improvement: existing knowledge is sound but not widely used, better results have been demonstrated in real-world settings, and current defect rates affect many patients somewhat, or at least a few patients profoundly.’* [4]

For this project the two initiatives have demonstrated real-world improvements in reducing SSI- and anaemia-related complications, both of which have profound effects on patients and their outcomes [28, 37]. However, these protocols are not widely used across the NHS.

### Faculty recruitment

*‘Five to 15 experts are identified in the relevant disciplines, including international subject matter experts as well as application experts, individual clinicians who have demonstrated breakthrough performance in their own practice. One expert is asked to chair the collaborative and is responsible for establishing the vision of a new system of care, providing faculty leadership, and teaching and coaching the participating teams.’ ‘The chair and the expert faculty assist in creating the specific content for the collaborative, including appropriate aims, measurement strategies and a list of evidence-based changes. An Improvement Advisor teaches and coaches teams on improvement methods and how to apply them in local settings.’* [4]

The CI and clinical lead are trained in Breakthrough Series methodology at IHI. The CI has used this approach to improve hip fracture care with six NHS organisations. The study team and collaborators include a number of recognised experts of the two relevant fields, namely prosthetic joint infection and pre-operative anaemia management. We will draw on their expertise to guide the development of the project and provide expert advice at learning events and in overcoming some barriers to local implementation of the initiatives.

### Enrolment of participating organisations and teams

*‘Organisations elect to join a collaborative through an application process, appointing multidisciplinary teams within the organisation charged to learn from the collaborative process, conduct small-scale tests of change, and help successful changes become standard practices. Senior leaders from participating organisations are expected to guide, support, and encourage the improvement teams, and to bear responsibility for the sustainability of the teams’ effective changes.’* [4]

For this study, calls for interested centres will be made via the British Orthopaedic Association (BOA) and via the sponsor, Northumbria Healthcare NHS Foundation Trust, with the help of NHS Improvement. This approach will target senior leaders, management and clinicians from each Trust to engage with, and be supportive of, the improvement process. If more than the required 40 Trusts are interested preference will be given to those performing greater numbers of hip and knee replacement procedures per year. Local teams will consist of a variety of healthcare professionals including orthopaedic surgeons, haematologists, microbiologists, anaesthetists, nurses and trainees. We will use existing surgical trainee research collaboratives to spread word of the study and encourage surgical trainee participation. The set-up phase will be used to reflect upon the methodology, build local engagement, enthusiasm and momentum and prepare the measurement and reporting framework for local performance data.

### Learning Sessions

*‘Traditional Learning Sessions are face-to-face meetings, usually three of which are conducted during a typical collaborative, bringing together multidisciplinary teams from each organisation and the expert faculty to exchange ideas. At the first Learning Session, the expert faculty present a vision for ideal care in the topic area and specific changes, called a Change Package, that, when applied locally, will improve significantly the system’s performance. Teams learn from an Improvement Advisor the Model for Improvement that enables teams to test these powerful change ideas locally, and then reflect, learn and refine these tests. At the second and third Learning Sessions, team members learn even more from one another as they report on successes, barriers and lessons learned in general sessions, workshops, storyboard presentations and informal dialogue and exchange. Formal academic knowledge is bolstered by the practical voices of peers who can say, “I had the same problem; let me tell you how I solved it”.’* [4]

For this study we will be undertaking three 1-day, face-to-face group Learning Sessions for each of the two arms of the trial. During these events we will teach participating Trusts the relevant protocol depending on their random trial allocation. We will also review the evidence, governance arrangements, business cases, communication strategies, pathways, data collection and reporting arrangements. These events will bring together experts, programme leads, improvement fellows, patient leaders and up to four study team members from each Trust to encourage collaborative learning and aid local implementation of the quality improvement measures. The learning events for the two groups will occur on separate dates to reduce the risks of contamination. At the end of the study period a further series of three learning events will be run so that Trusts can be taught the second protocol. This is intended to improve compliance, minimise crossover and maximise the benefit to Trusts in being involved in the trial.

### Action Periods

*‘During Action Periods between the Learning Sessions, teams test and implement changes in their local settings — and collect data to measure the impact of the changes. They submit monthly progress reports for the entire collaborative to review, and are supported by conference calls, peer site visits and web-based discussions that enable them to share information and learn from national experts and other healthcare organisations. The aim is to build collaboration and support the organisations as they try out new ideas, even at a distance.’* [4]

For this study, between Learning Sessions, teams will test and implement changes in their local settings and collect data to measure the impact change, supported by learning and quality accounts. The Model for Improvement will be applied as a way of testing small-scale improvement cycles. Experience of this work has taught us that reliable and regular measuring of impact change together with providing timely feedback to local teams has been key to successful implementation.

A bespoke, secure, electronic data-extraction system will be developed for this study. This will assist in the production of monthly progress reports. An information technology (IT) support line will be in place, provided by e-Dendrite, our IT partner, for any IT issues. Study team members will also be contactable for any advice as required throughout the study period and will explore various ways to encourage communication and collaboration between teams. The learning events will act as networking opportunities for Trusts to form working, collaborative relationships and contacts.

### Summative congresses and publications

*‘Once the collaborative is complete, the work is documented and teams present their results and lessons learned to individuals from non-participating organisations at national and international conferences and meetings.’* [4]

For this study, a summative session will be held on completion of the implementation of the protocols for each randomised arm. A second will be held after Trusts have had the opportunity to introduce the alternate protocol, to which all collaborators will be invited. The study results are due to be presented at the BOA National Congress 2020 and publication in a high-impact journal will be sought. There are potential links for the study results to influence best practice tariffs and national guidelines.

### Measurement and evaluation

*‘Collaboratives involve regular measurement and assessment. All teams are required to maintain run charts tracking their system measures over time and key faculty members review each team’s monthly report to assess the overall progress of the collaborative.’* [4]

The same bespoke, secure, electronic data-extraction system will also provide near to live feedback to teams on their Trust’s improvement journey. The system will automatically generate a number of metrics and run charts mapping the individual teams’ and whole collaboratives’ improvement journey over time. An example would be mapping the anaemia-screening rate per month over time. This data can be used by the teams and select faculty members.

### Outcomes and measures

The trial will include quantitative and qualitative outcome measures. The aim of this mixed-methods approach is to provide a detailed and robust evaluation of the improvement collaboratives, addressing some of the previously reported deficiencies in collaborative research. To account for variation in clinical practice for issuing blood transfusions between centres, the blood transfusion policy from each will be collated and compared.

### Quantitative outcomes and measures

We will collect patient-level outcomes and process measure outcomes. Both arms of the trial must collect all of the same patient-level outcomes to allow comparison between the two groups as they are acting as each other’s control.

#### Patient-level outcomes

- Blood transfusion within 7 days before surgery or 7 days after surgery (primary outcome for QIST: Anaemia)
- Deep SSI (MSSA) up to 90 days post surgery (using the Public Health England (PHE) definition at 90 days, not 1 year) (primary outcome for QIST: Infection)
- Deep SSI (any causative organism) and name of pathogen (if known)
- Superficial infection up to 30 days post surgery, and name of pathogen (if known)
- Length of hospital stay in days (number of midnights spent in hospital)
- Readmission to hospital within 30 days of discharge
- Critical care admission within 30 days of surgery (regardless of previous discharge)
- Time spent in critical care within 30 days of surgery (number of midnights spent in the unit)

#### Process measures for QIST: Anaemia

- Date patient screened for anaemia
- Pre-operative blood results: haemoglobin (g/L), ferritin (μg/L), estimated glomerular filtration rate (eGFR) (mL/min/1.73 m^2^)
- Protocol functionality (follows Trust-agreed protocol)
- Was iron given to the patient?
- If iron is given was this orally and/or intravenously administered?

#### Process measures for QIST: Infection

- Date patient screened for MSSA
- Results: ‘MSSA positive’ or ‘MSSA negative’ or ‘Not tested’
- Protocol functionality (follows Trust-agreed protocol)
- Date decolonising pack dispensed
- Confirmation used by the patient: Yes/No

In addition, patient comorbidity data will be collected and presented in the description of surgical patients seen at Trusts, and used in the economic evaluation.

### Verification of surgical site infections

#### Identification

Identification of SSIs in the study population will rely upon local follow-up measures, which may improve by being part of the collaborative and local reporting by orthopaedic trainees as part of the National Collaborative Orthopaedic Research Network. Orthopaedic trainees will have an understanding of SSI from their clinical training. This will be built upon with training around recording and auditing data collected for the QIST trial. As an incentive to report infections, etc., contributing trainees will be included as collaborators on the outputs from the trial.

Upon indication of any potential SSI, based on the data entered into the QIST database, the recruiting site will be contacted. If the treating clinical team diagnosed a ‘deep infection’, prompt diagnosis and treatment of this infection is fundamental to the patient’s routine clinical care, so this will always be documented in the patient’s medical record. Site staff will be asked to review the patient’s medical records to provide additional information. The site will be asked to provide completely redacted (hospital number only) copies of relevant medical notes and any *re-operation records* for surgery related to the index hip or knee arthroplasty; *microbiology reports* if samples of the suspected infected tissues around the hip/knee were sent for analysis; and/or *imaging reports* for any deep imaging that occurred in relation to suspected infection. These data will be collated by the trial team in York.

All Trusts should be able to reliably identify patients who are readmitted to the same hospital within 90 days of surgery. However, specialist centres that are likely to perform a high proportion of operations take their patients from a wide area and are, therefore, not guaranteed to know whether patients are subsequently admitted to a different, possibly more local hospital following surgery. We will, therefore, request readmission data from NHS Digital via Hospital Episode Statistics (HES) data.

#### Classification of SSI

Our initial intention was to use the Centres for Disease Control (CDC) classification of infections, for which the cut-off is 90 days. However, a number of Trusts are already collecting infection data for PHE and are, therefore, more familiar with the PHE classification than the CDC’s.

**Fig. 1:**
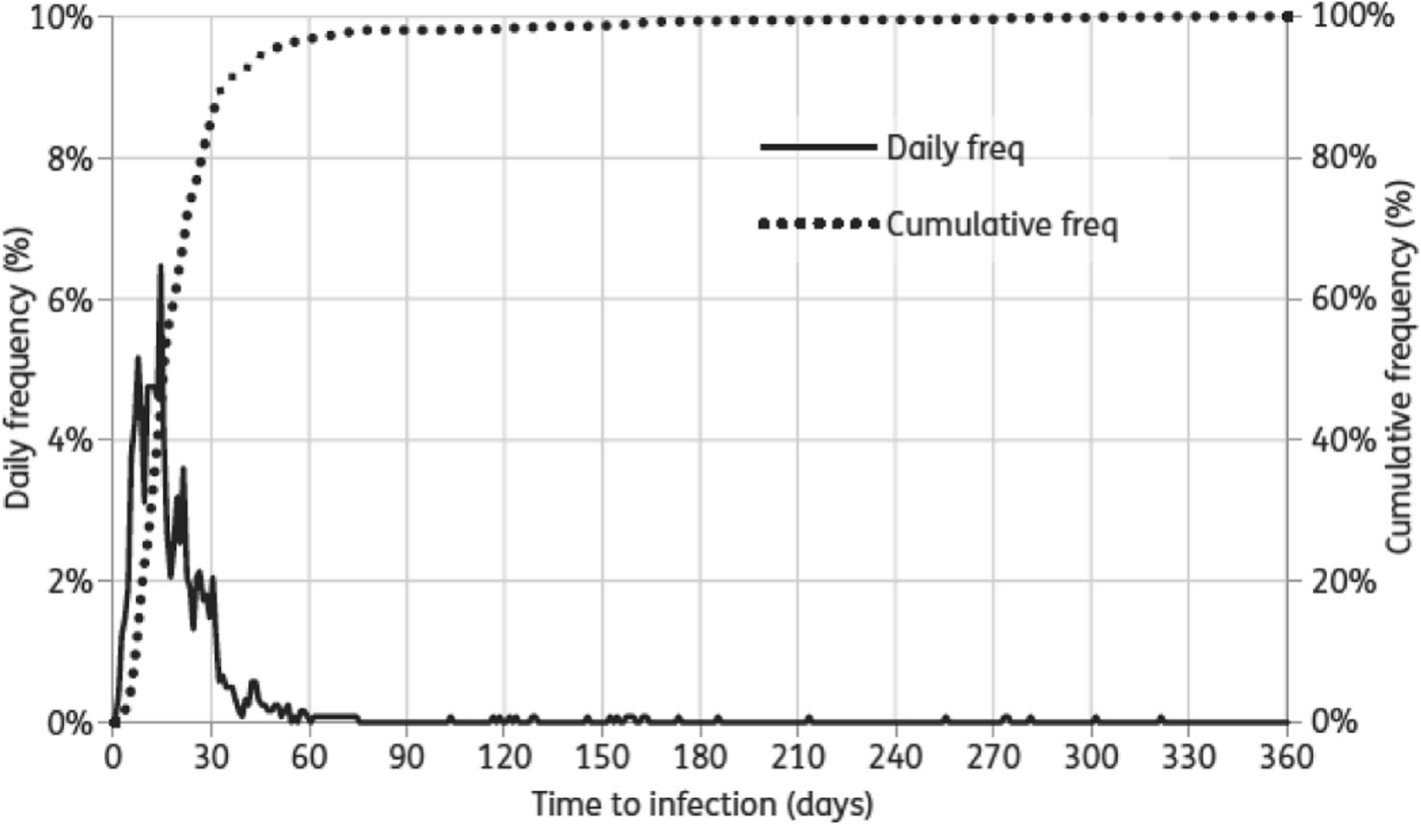
Public Health England (PHE) chart showing when a diagnosis of surgical site infections is made. Reproduced from Theresa Lamagni. Epidemiology and burden of prosthetic joint infections. Journal of Antimicrobial Chemotherapy 2014;69 Suppl_1: i5–i10, doi: 10.1093/jac/dku247. By permission of Oxford University Press on behalf of the British Society for Antimicrobial Chemotherapy. This figure is not included under the Creative Commons license of this publication. For permissions, please contact journals.permissions@oup.comPlease visit: https://academic.oup.com/jac/article/69/suppl_1/i5/772200?searchresult=1

In discussion with the PHE Surgical Site Infection Surveillance Team it was found that there were only subtle differences between the CDC and PHE classifications. The main difference is in the time frame: 90 days for CDC and 1 year for PHE. PHE has provided data that demonstrate that using the PHE classifications and cutting the data at 90 days (as for CDC and as planned for this trial) would not make a significant difference in identifying post-operative infections in our population (Fig. 1).

**Fig. 2:**
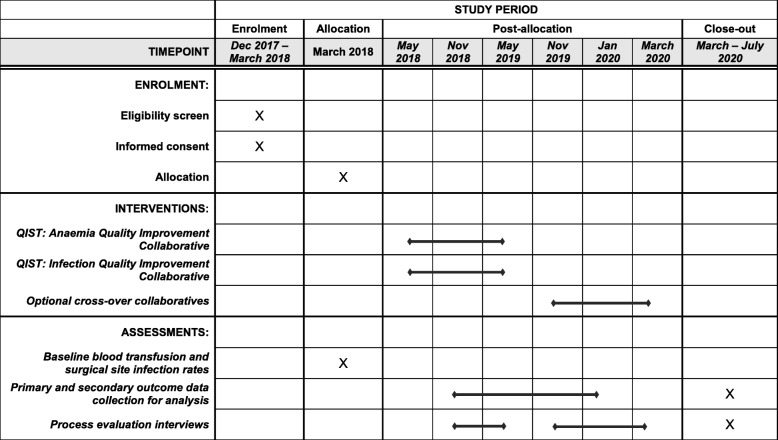
Standard Protocol Items: Recommendations for Interventional Trials (SPIRIT) Figure for the Quality Improvement for Surgical Teams (QIST) trial

It has, therefore, been agreed that the PHE classification will be used by Trusts and the primary endpoint for infection will remain at 90 days [46].

#### Confirmation of SSI

To confirm the robustness of the above reporting system for identifying SSIs, an Independent Outcome Classification (IOC) Group will be convened. This group will be comprised of consultant revision hip and/or knee surgeons and consultant microbiologists. The IOC Group will review all cases deemed to have a SSI (deep or superficial, MSSA or other or unknown causative organism) as well as a purposeful sample of 50 cases where no infection was reported. The IOC Group will be given access to all the data collected by the research team as well as relevant, redacted sections of the patient’s medical records.

The IOC Group will use both the CDC and PHE classifications when reviewing cases to ensure that the reporting of deep infection outcomes can be presented to the wider international audience.

It is important to note that it is not uncommon for the diagnosis of infection to be made in the absence of positive microbiological findings. This may be due to the concurrent administration of antibiotics for other infections, e.g. chest, or due to sampling error, or the bacteria being hard to culture.

The medical records of those patients who die before their primary outcome can be assessed will be reviewed by the site team to understand whether SSI may have had a contributory role to their death. It is expected that this will be rare in our cohort and for the vast majority this will not be the case, as most early deaths are related to cardiovascular or respiratory events.

### Qualitative process evaluation

The process evaluation will adopt a qualitative approach to both the formative and summative elements of the evaluation of support. The formative evaluation will help the project team to review and/or modify the improvement/support plan should feedback indicate this is necessary. The summative element of the evaluation will show where and whether the improvement programme has been successful and met its objectives in the way intended. The main evaluation questions are:

1. What has happened during the improvement programme?
2. Were the various learning activities successful and if so why?
3. Tell me about the support you have received
4. What has been the effect of being part of a BSC?

These will guide the choice of more specific evaluation questions.

The learning events will be observed by the evaluator and 80 semi-structured telephone interviews held with team members from the participating Trusts throughout the study. At the launch event teams will be given written information about this evaluation.

#### Intervention delivery phase (November 2018 – April 2019)

Telephone interviews will be held with two members of each of 10 teams (the project lead and one other team member) in each trial arm (20 teams in all, 40 interviews).

#### Follow-up phase (November 2019 – March 2020)

Telephone interviews will be held with two members of each of 10 teams in each intervention arm (the project lead and one other team member) in each trial arm (20 teams in all, 40 interviews).

The second team member will be purposively selected from those who volunteer, to reflect a spread of the various health professionals involved in the quality improvement initiatives (e.g. pre-assessment nurses, anaesthetists, ward nursing staff, medical staff, infection control staff).

Verbal consent to recording the interviews and the use of unattributed quotes will be sought prior to the interviews. Recording will be password-protected and encrypted. Once transcribed, all recordings will be destroyed, and only the anonymised transcripts retained. Throughout the process the constant comparative method will be used to identify commonalities and emerging patterns and themes across sites.

This methodology and the evaluation questions should enable a broad spread across all sites and provide rich data to facilitate understanding of why and how using BSCs as a quality improvement initiative are and are not successful and may help to explain why the ‘same’ quality improvement initiatives may be implemented and received in different ways in different sites.

## Economic evaluation

The economic evaluation aims to assess the cost-effectiveness of the quality improvement initiative for MSSA and anaemia detection prior to elective total hip and knee replacement. The objectives of our analyses are to determine (1) the costs associated with the set-up, administration and delivery of both improvement initiatives; (2) whether the improvement initiatives lead to cost savings in terms of NHS healthcare resource use; and (3) whether the improvement initiatives lead to further benefits in terms of patients’ improved health. The analyses will be conducted from the perspective of the NHS and for the duration of the trial.

The economic costing of the quality improvement initiative represents the primary objective of this evaluation. The costs will be analysed from a NHS and Personal Social Services (PSS) perspective. We will consider all resource use during the set-up, process administration and delivery of the quality improvement initiative. A resource-use survey will be specifically designed for the QIST trial to capture a comprehensive list of inputs associated with the quality improvement initiative both at Trust and patient level. Both staff and non-staff or material costs (e.g. equipment investments, information technology and consumables) will be considered in the analysis. Staff time associated with the delivery of the programme will be valued using national unit costs per working hour for each Agenda for Change band of staff. Unit costs for the analysis will be derived from established national costing sources such as NHS Reference Costs, PSS Research Unit costs of health and social care, and the *British National Formulary*.

Secondary outcomes for the trial will be used to assess the cost-savings associated with the qualitative improvement initiative (e.g. hospital readmission, length of hospital stay, readmissions and critical care admission, regardless of previous discharge). Besides, transfusions and infection rates will be used to assess the cost-effectiveness of the improvement initiatives. Sensitivity analyses will be undertaken to explore and quantify any uncertainty around economic estimates.

## Analysis and reporting

A detailed statistical analysis plan for the analysis of quantitative data will be prepared and signed off by the Trial Steering Committee prior to data analysis. Analysis will be conducted using the principles of intention to treat, i.e. patients and Trusts will be analysed in the group to which they were randomised, irrespective of whether or not they actually received, or adhered to, their allocated quality improvement initiative. Further per-protocol and case analyses will also be performed. Statistical significance will be assessed using two-sided tests at the 5% level. The flow of NHS Trusts and patients/procedures through the trial will be presented in a Consolidated Standards of Reporting Trials (CONSORT) flow diagram [47].

The primary analyses will, separately, compare transfusion and deep infection rates between the quality improvement initiative and control groups at the procedure level using a mixed-effects logistic regression model. The models will adjust for pertinent baseline covariates at the patient/procedure level (e.g. procedure type, age, gender of patient) and at the Trust level (e.g. the minimisation factors used in the randomisation), with NHS Trust as a random effect. Adjusting for baseline factors likely to be predictive of outcome, e.g. procedure type, will increase the precision of the estimated treatment effects. The exact model specifications will be agreed prior to the completion of data extraction, and pre-specified in the statistical analysis plan. We shall analyse at the level of the procedure, rather than the patient, since it is possible that a small number of patients will undergo more than one eligible procedure during the study period. We will treat the procedures as independent, and conduct sensitivity analyses retaining only the earliest procedure for each patient to assess the impact of duplicate patients on the results. We anticipate that any impact will be minimal. The secondary outcomes of superficial infection, hospital readmission, length of stay and critical care admission will be analysed using appropriate regression techniques based on the type of data.

Data on processes (e.g. compliance with elements of each of the quality improvement protocols such as proportion of eligible patients tested for MSSA; proportion of decolonising packs dispensed; proportion screened for anaemia; proportion of patients with anaemia treated before surgery) will be summarised descriptively by treatment group.

Analyses and results will be reported in accordance with the CONSORT extension for cluster trials [47].

### Project data portal

A bespoke data portal for the project will be developed within the secure N3 NHS network. e-Dendrite is an NHS-verified supplier, and it currently provides national audit tools for all NHS Trusts in England and, therefore, is considered a trusted third party.

Fully anonymised aggregated data will be fed back to each Trust via the Quality Improvement Team, on a monthly basis.

A fully anonymised dataset of historical Patient Administration System (PAS) data for each Trust for the 12 months prior to the intervention period will be sent by secure means to a secure server at York Trials Unit (YTU). A fully anonymised patient dataset and Trust-level aggregated dataset at the first extraction month after the start of the trial will be supplied to YTU to check the quality of data and data collection. A fully anonymised patient-level dataset and Trust-level aggregated dataset will be sent to YTU’s secure server at the end of the intervention period.

## Patient and public involvement

We sought the views of patients and the public on the use of health information without explicit patient consent for this study. Members of a total hip replacement patient representative group (THUG) were unanimously comfortable with hospitals being randomised as a Trust and not obtaining individual patient-level consent. Real-time feedback from 16 inpatients gave unequivocal support for health information being used in this way.

The Trial Steering Committee membership will include a patient representative. The THUG group have agreed to remain involved and will be given regular updates on progress and asked to comment on relevant documentation as appropriate.

In addition, a patient representative will be included in the co-production of the Learning Sessions, contributing to both the development and the delivery of the sessions.

## Ethics and governance

The study is sponsored by the Northumbria Healthcare NHS Foundation Trust.

Written information on the qualitative evaluation will be distributed to NHS staff at the first learning events. Verbal consent will be sought from NHS staff prior to recording the qualitative interviews. Once transcribed, all recordings will be destroyed, and only the anonymised transcripts retained. Interviews will be recorded and transcribed verbatim. Verbal permission will also be sought from interviewees to use unattributed quotes in reports and/or publications resulting from this programme.

Patient-level consent will not be sought as the introduction of the anaemia and infection screening procedures are quality improvement initiatives and both have been demonstrated to be effective and recommended for use across the NHS [10, 25].

The intervention is targeted at Trust level and NHS research ethics approval is not required. Institutional approval was sought from the Health Sciences Research Governance Committee of the University of York; the committee felt that research ethics approval was not necessary for this study (reference no: HSRGC/2018/256/D). HRA approval was sought and obtained (IRAS 238457).

An application to the Confidential Advisory Group is not necessary as the only identifier to be used when transferring data from Trusts to e-Dendrite will be the local Hospital Number. Data passed from e-Dendrite to the Quality Improvement Team and to YTU will be fully anonymised. Within each of the Trusts, data will be collected by staff members who already have permission to access the patient data as part of their role.

The trial will comply with the principles of the Declaration of Helsinki, and be conducted in accordance with the principles of Good Clinical Practice. This protocol has been reported in accordance with SPIRIT guidelines (Fig. 2).

## Discussion

Successful implementation of pre-operative anaemia optimisation for elective hip and knee replacement patients has been achieved and positive results reported from at least one NHS Trusts in England [28]. This work showed that a relatively straightforward clinical pathway including the use of orally administered and/or IV iron resulted in significant reductions in the number of patients transfused (6 v 4.1% *p* = 0.005), length of hospital stay (3.9 v 3.6 days *p* = 0.017), critical care admissions (1.27 v 0.55% *p* = 0.03) and hospital readmissions (4.5% v 3.0% *p* = 0.02). This was found to be cost-effective, resulting in savings of £162.46 per patient screened, or £406,000 for a Trust performing 2500 primary THR or TKRs per annum.

Similarly, a relatively simple programme for pre-operative MSSA screening and decolonisation has also been successfully implemented in an English NHS Trust for elective hip and knee arthroplasty patients [37]. This resulted in a significant reduction in the rate of MSSA prosthetic joint infection (0.75 v 0.25% *p* < 0.0001). This programme is reported to have prevented 47 MSSA prosthetic infections and cost £1893 per infection avoided, each of which would have cost tens of thousands of pounds to treat.

It is expected that Trusts will see similar benefits from anaemia and MSSA screening programmes by being part of the QIST trial.

The scale of the proposed project brings considerable challenges and potential barriers to delivery [48]. We expect these challenges to include recruiting and sustaining 40 organisations, each with its own culture and context, and dealing with multiple stakeholders across a large geographical area. The project will be managed by a strong team with a proven track record including: project leadership at a senior level, advanced project management and governance and supported by an experienced research team at YTU. The participatory, collaborative implementation methodology of the BSC model provides the opportunity for flexibility in implementation to reflect contexts of individual participating Trusts.

## Trial status

At the time of submission, we are working to Protocol Version 1.5 dated 18 March 2019. Recruitment began in October 2017 and was completed May 2018 when 41 Trusts were randomised.

## Data Availability

Not applicable.

## Abbreviations

BOA: British Orthopaedic Association
BSC: Breakthrough Series Collaborative
CDC: Center for Disease Control
CI: Chief investigator
HES: Hospital Episode Statistics
ICC: Intraclass correlation coefficient
IHI: Institute for Healthcare Improvement
IOC: Independent Outcome Classification
IT: Information technology
IV: Intravenously administered
MSSA: Methicillin Sensitive *Staphylococcus aureus*
NHS: National Health Service
NJR: National Joint Registry
PAS: Patient Administration System
PHE: Public Health England
PI: Principal investigator
PSS: Personal Social Services
QIC: Quality Improvement Collaborative
QIST: Quality Improvement for Surgical Teams
RCT: Randomised controlled trial
*S. Aureus*: *Staphylococcus aureus*
SSI: Surgical site infection
TJR: Total joint replacement
UK: United Kingdom
YTU: York Trials Unit
